# A population-based study examining the emergence of community-associated methicillin-resistant *Staphylococcus aureus *USA300 in New York City

**DOI:** 10.1186/1476-0711-5-29

**Published:** 2006-11-30

**Authors:** Simona Bratu, David Landman, Jyoti Gupta, Manoj Trehan, Monica Panwar, John Quale

**Affiliations:** 1 Division of Infectious Diseases, State University of New York – Downstate Medical Center, Brooklyn, New York, USA

## Abstract

**Background:**

Community-associated methicillin-resistant *Staphylococcus aureus *(CA-MRSA) is a serious pathogen in several regions in the United States. It is unclear which populations are at high risk for the emergence of these strains.

**Methods:**

All unique patient isolates of *S. aureus *were collected from hospitals in Brooklyn, NY over a three-month period. Isolates of MRSA that were susceptible to clindamycin underwent SCC*mec *typing. Isolates with the SCC*mec *type IV (characteristic of CA-MRSA strains) underwent ribotyping. Demographic information involving the neighborhoods of Brooklyn was also gathered and correlated with the prevalence of CA-MRSA strains.

**Results:**

Of 1316 isolates collected during the surveillance, 217 were MRSA susceptible to clindamycin. A total of 125 isolates possessed SCC*mec *type IV; 72 belonged to the USA300 strain and five belonged to the USA400 strain. Hospitals in the eastern part of the city had the highest prevalence of USA300 strain. Individuals in the eastern region, when compared to the western region, were more likely to be Black, Hispanic, female, and < 18 years of age, and to have households of ≥ 3 persons. In addition, the median household income was lower, and the proportion of individuals on public assistance was higher, for the population in the eastern region.

**Conclusion:**

The USA300 strain of CA-MRSA is emerging in New York City. In this population-based study, urban regions of lower socioeconomic status and with evidence of overcrowding appear to be at higher risk for the emergence of this pathogen.

## Background

Community-associated methicillin-resistant *Staphylococcus aureus *(CA-MRSA) has emerged as a frequent and serious pathogen in several regions in the United States. The CA-MRSA strains have distinctive phenotypic and genotypic features when compared to typical hospital-acquired strains. Most CA-MRSA remain susceptible to other non-β-lactam antibiotics [1-4]. CA-MRSA strains typically possess type IV SCC*mec *gene and the Panton-Valentine leukocidin (PVL)[1,4,5]. Two distinctive pulsed field gel electrophoresis types of CA-MRSA have predominated in the United States [1]. The USA400 type was isolated from children in the Midwestern United States, and has been associated with nosocomial infections in neonates and post-partum women [6-8]. The USA300 type has been associated with outbreaks in prisons and sports teams, and has become the predominant type in certain regions in the United States [2,9,10].

Prior to the emergence of the USA 300/400 strains, most patients with community-onset MRSA infections had identifiable risk factors, including recent hospitalization or nursing home residence, invasive/percutaneous procedure, and/or chronic dialysis therapy [11-13]. However, initial reports have noted patients with USA300/400 strains have not possessed these risk factors; risk factors for infection with these strains remain poorly defined.

Most reports of CA-MRSA have examined outbreak situations; relatively few studies have performed population-based analyses [14]. In this report, we examine the prevalence of CA-MRSA in Brooklyn, NY and examine characteristics of urban neighborhoods identified with a higher prevalence.

## Materials and methods

### Surveillance study

From December 2005 through February 2006, all single patient isolates of *S. aureus *were gathered from 15 of the 16 hospitals in Brooklyn, NY; the Department of Veterans Affairs Medical Center, which serves select patients from throughout the city, was not included in the study. Bacterial isolates were identified by the participating microbiology laboratories according to standard techniques. Susceptibility testing was performed in the central research laboratory by the agar or broth (for tigecycline and daptomycin) dilution methods, according to CLSI standards [15].

### Characterization of bacterial isolates

Since susceptibility to clindamycin and possession of SCC*mec *IV are typical features of the USA 300/400 strains, all MRSA isolates gathered in the surveillance study that were susceptible to clindamycin underwent initial *mec *typing according to the methods of Oliveira et al [16]. Isolates that were nontypeable or found to possess a SCC*mec *IV underwent further *mec *characterization according to the multiplex assay of Zhang et al [17]. Selected isolates also underwent ribotyping, pulsed field gel electrophoresis, and PCR screening for the genes encoding PVL, as previously described [1,8].

### Population-based analysis

Data concerning the city of Brooklyn, and the 72 neighborhoods that comprise the city, were obtained using the Infoshare Community Data System (Community Studies of New York, Inc). Demographic, income, and health data were recorded for each of the neighborhoods. The information in this database largely reflects the year 2000 census records. The 72 Brooklyn neighborhoods were assigned, based on location, to one of the 15 hospitals as the primary medical center delivering care to the neighborhood.

A retrospective chart review was conducted on selected patients; information collected included demographic data (including home address), record of recent hospitalization, clinical status on presentation, and clinical outcome (survival).

Statistical analysis included chi square analysis for categorical data and student's *t*-test for continuous variables.

This study has been approved by the Institutional Review Board at SUNY- Downstate Medical Center.

## Results

A total of 1316 isolates of *S. aureus *were collected during the three-month surveillance study; 581 (44%) were found to be MRSA (Table 1). Of the MRSA isolates, 217 (37%) were susceptible to clindamycin. SCC*mec *type IV was found in 125 (58%) of these isolates (123 with type IVa and two with type IVb). One isolate possessed SCC*mec *type I, 47 possessed type II, and 44 were unable to be typed. Seventy-five (60%) of the isolates with SCC*mec *type IV carried the genes for PVL. Ribotyping was performed on 120 of the 125 (96%) isolates with SCC*mec *type IV, and 72 (58%) belonged to the USA300 strain. PVL genes were identified in 81% of the USA300 isolates. Only 5 (4%) isolates belonged to the USA400 strain. The remaining 48 isolates belonged to 12 different ribogroups.

**Table 1:** Overall susceptibility results of 1316 *Staphylococcus aureus *isolates collected in the city-wide surveillance study.

	MIC_50_	MIC_90_	Range	Susceptible
	μg/ml			

Oxacillin	0.5	>4	≤.06–>4	56%
Azithromycin	>8	>8	≤0.25–>8	33%
Clindamycin	0.06	>4	≤.03–>4	66%
Vancomycin	0.5	1	≤0.25–>1	100%
Ciprofloxacin	4	>4	≤.06–>4	49%
Daptomycin	0.25	0.5	≤0.12–1	100%
Tigecycline	0.06	0.25	≤0.015–0.5	100%
Trimethoprim-sulfamethoxazole	≤0.5	≤0.5	≤0.5–>4	96%

**Figure 1:**
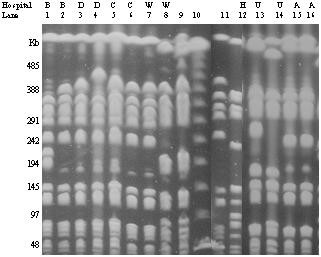
Pulsed field gel electrophoresis results for selected MRSA isolates. Lanes 1–7: clinical isolates belonging to the same ribotype as USA300. Lane 8: Clinical isolate belonging to the same ribotype as USA400. Lane 9: representative USA400 strain. Lane 10: lamda ladder. Lane 11: representative USA300 isolate. Lanes 12–16: isolates with nontypeable SCC*mec*.

Fingerprinting by pulsed field gel electrophoresis correlated well with the ribotyping results. Representative isolates belonging to the same ribogroup as USA300 also had the same pulsed field type (Fig. 1). To assess if bacteria with a nontypeable SCC*mec *were unrecognized strains related to either USA300 or USA400 types, seven nontypeable isolates underwent pulsed field gel electrophoresis. None of these isolates were closely related to the two CA-MRSA strains (Fig. 1).

The 72 isolates belonging to the USA300 type were examined in further detail. Forty-six isolates originated from wound/soft tissue cultures, seven were from respiratory specimens, seven were from blood cultures and 12 cultures were from miscellaneous or unidentified sources. Fifty-one of the 72 (71%) isolates originated from patients from six hospitals; however, these hospitals supplied 49% of all *S. aureus *isolates (P < 0.001). The USA300 strains accounted for 7.9% (range, 6.7–9.2%) of the *S. aureus *isolates collected from these six hospitals. In contrast, the USA300 strains accounted for 3.1% (range 0–5.0%) of the *S. aureus *isolated from the remaining nine hospitals.

**Figure 2:**
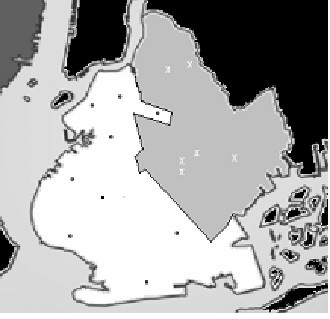
Map of Brooklyn indicating regions with low prevalence (white area) and high prevalence (gray area) for *S. aureus *USA300 strain. Black circles and white X's represent medical centers in the low and high prevalence regions, respectively.

The six hospitals with the greater prevalence of USA300 strains all serve neighborhoods located in the eastern section of the city, while the nine remaining hospitals serve neighborhoods in the western half of the city (Fig. 2). During the surveillance period, there were 4.6 cases/100,000 in the high prevalence region, compared to 1.6 cases/100,000 in the lower region. The populations comprising these two regions displayed markedly different characteristics (Table 2). The population in the high prevalence region was more likely to be Black and Hispanic, female, and less than 18 years of age. Residents in the high prevalence region were more likely to be economically disadvantaged, to have ≥ 3 persons per household, and had a nearly sevenfold increased incidence of newly diagnosed HIV infection.

**Table 2:** Comparison of neighborhoods with low and high prevalence rates for *S. aureus *isolates belonging to the USA300 clone.

Region characteristic	Western (low prevalence) neighborhoods	Eastern (high prevalence) neighborhoods	
White	61.4%	19.0%	P < 0.001
Black	14.7%	60.1%	P < 0.001
Asian	12.1%	2.5%	P < 0.001
Hispanic	17.1%	24.1%	P < 0.001
Female	50.8%	54.4%	P < 0.001
Age < 18 years	22.8%	30.6%	P < 0.001
Residents Medicaid eligible	31.5%	41.3%	P < 0.001
Residents on Public Assistance	3.5%	8.5%	P < 0.001
Households with ≥ 3 persons	41.1%	51.3%	P < 0.001
New HIV diagnoses	9.1 cases per 100,000	61.7 cases per 100,000	P < 0.001
Average household income (Mean ± SD)	$45,435 ± 16,132	$30,477 ± 9,461	P < 0.001

**Figure 3:**
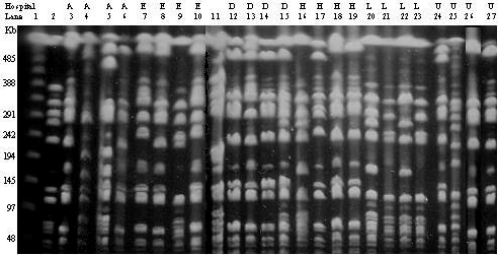
Pulsed field gel electrophoresis of clindamycin-resistant clinical isolates. Lane 1: lamda ladder. Lane 2: representative USA300 isolate. Lanes 3–10: isolates collected from two hospitals in the western part of the city. Lane 11: representative USA400 isolate. Lanes 12–15: isolates from a hospital in the eastern part of the city. Lanes 16–27: isolates collected from three hospitals in the western part of the city.

To determine if our selection criteria (clindamycin-susceptible MRSA) was too restrictive for identifying the USA300 strains, pulsed field gel electrophoresis was performed on the first four clindamycin-resistant isolates from six hospitals. To examine for potential bias, 20 isolates originated from hospitals in the western (low prevalence) part of the city. None of clindamycin-resistant MRSA were related to either USA300 or USA400 strains (Fig. 3).

To determine if patients with cultures positive for the USA300 strain were representative of the population of the high prevalence neighborhoods, records of 20 patients from two medical centers within the higher prevalence region were reviewed. Of the 20 patients, three were ≤ 18 years of age and 11 were female. Seventeen of the 20 patients resided within the neighborhoods with the higher prevalence. Two patients were known to be HIV positive. Prior hospitalization (within the previous year) was documented in five patients and one patient was on hemodialysis. Six patients had Medicaid or Medicare as their health insurance, and only two patients possessed private health insurance. Race and ethnicity were recorded in only ten of the patients; nine were Black and one was white/Hispanic.

## Discussion

Several studies, often performed without the benefit of genetic fingerprinting of the bacterial isolates, found several identifiable risk factors (e.g., hospitalization within one year, nursing home residence, hemodialysis, or placement of a long-term intravascular device) that were associated with community-onset MRSA infection or colonization [11-13]. However, this scenario has changed dramatically with the emergence of two MRSA strains, USA300 and USA400. While several well-described outbreaks involving USA300 (e.g., in prisons and sports teams) and USA400 (e.g., in postpartum women and maternity units) have been reported [7-10], risk factors for acquisition of these strains in the general population are largely unknown. In Atlanta, patients with skin and soft tissue infections with the USA300/400 strains were more likely to be black and female when compared to patients with infections due to MSSA [2]. The USA300 type predominated in this study, and the medical center served a largely black and indigent population [2]. In Minnesota, patients with cultures with CA-MRSA were more likely to be younger, nonwhite, and of lower socioeconomic status when compared to patients with hospital-acquired strains of MRSA [4]. In a multicenter study involving patients from Atlanta, Baltimore, and Minnesota, patients with CA-MRSA were likely to have several underlying conditions (e.g., tobacco use, prior skin infections, diabetes mellitus, asthma, and HIV infection) and were of lower socioeconomic status; isolates in this report were not fingerprinted [14]. In a nationwide survey examining rates of nasal colonization, *S. aureus *was more common in men, those with asthma, and in subjects < 65 years of age; blacks and Mexicans had lower colonization rates when compared to whites. Risk factors for MRSA colonization included age > 65 years, female sex, underlying diabetes mellitus, and residence in a long-term care facility; Hispanics were less likely than whites to be colonized with MRSA [3]. However, approximately half of the MRSA isolates in the last study possessed SCC*mec *II, suggesting that many were hospital-associated strains.

As the boundary between cases with nosocomial and community-associated MRSA becomes hazy, it is increasingly apparent that future epidemiological studies will require thorough characterization of the bacterial isolates. In this report, only 35% of our isolates with the antibiotic phenotype suggestive of CA-MRSA (MRSA susceptible to clindamycin) belonged to the USA300/400 types. In addition, only 62% of isolates with SCC*mec *type IV belonged to the USA300/400 types; whether the other isolates represent CA-MRSA strains unique to our region requires further investigation.

In this report, we performed a population-based analysis of CA-MRSA in Brooklyn, NY using all *S. aureus *isolates identified in hospital microbiology laboratories. By itself, Brooklyn would rank as the fourth largest city in the United States, and has an extremely heterogeneous population. In this urban setting, we found a higher prevalence of USA300 strains in neighborhoods with several distinguishing characteristics. Neighborhoods with a higher prevalence of USA300 had a greater proportion of blacks, Hispanics, females, and children, and had measures indicative of a disadvantaged socioeconomic status. As more households had ≥ 3 persons in the high prevalence neighborhoods, crowded living conditions are likely an important contributing factor for the spread of the USA300 strain. Although racial and ethnic risk factors have been noted in other studies of CA-MRSA [2-4], it remains to be determined if these features are causal in nature or just reflect lower socioeconomic status (and crowded living conditions).

Our results are in stark contrast to a prior study examining epidemiology of *Streptococcus pneumoniae *in Brooklyn [18]. In that report, the western region of the city (identified with the lower prevalence of USA300) had a higher rate of penicillin-resistant *S. pneumoniae*, and was attributed to greater access to healthcare (and antimicrobial agents). Indeed, increased antibiotic consumption has been postulated as a protective factor against CA-MRSA in certain populations [3]. It is evident that in a large urban setting, these two resistant community pathogens do not share similar epidemiological characteristics.

## Conclusion

The USA300 strain of CA-MRSA is emerging in Brooklyn, NY. In this population-based study, urban regions with characteristics of lower socioeconomic status and with evidence of overcrowding appear to have a higher prevalence of this pathogen.

## Competing interests

The author(s) declare that they have no competing interests.

## Authors' contributions

SB, DL, and JQ conceived the study, participated its design and coordination, and helped draft the manuscript. JG, MT, and MP participated in the design and coordination of the study. All authors read and approved the final manuscript.
